# An overview of the effects and mechanisms of m6 A methylation on innate immune cells in sepsis

**DOI:** 10.3389/fimmu.2022.1041990

**Published:** 2022-11-24

**Authors:** Weiwei Qian, Yu Cao

**Affiliations:** ^1^ Emergency Department, Shangjinnanfu Hospital, West China Hospital, Sichuan University, Chengdu, Sichuan, China; ^2^ Emergency Department of West China Hospital, Sichuan University, Chengdu, Sichuan, China

**Keywords:** sepsis, m^6^A methylation, immune cells, inflammation, oxidative stress

## Abstract

**Introduction:**

Sepsis is a severe clinical syndrome caused by dysregulated systemic inflammatory responses to infection. Methylation modification, as a crucial mechanism of RNA functional modification, can manipulate the immunophenotype and functional activity of immune cells to participate in sepsis progression. This study aims to explore the mechanism of N6-methyladenosine (m6A) methylation modification in immune cell-mediated sepsis through keyword search.

**Methods:**

Literature retrieval.

**Results and Discussion:**

Literature retrieval reveals that m6A methylation is implicated in sepsis-induced lung injury and myocardial injury,as well as sepsis-related encephalopathy. Furthermore, it is found that m6A methylation can regulate sepsis by inhibiting the chemotaxis of neutrophils and the formation of neutrophil extracellular traps and suppressing macrophage phagocytosis, thereby playing a role in sepsis.

## 1 Introduction

Sepsis, as one of the most common complications in patients who undergo severe trauma, serious burn, shock, infection, or major surgery, is defined as potentially fatal organ dysfunction caused by dysregulated host systemic inflammatory and immune response to infection, mainly manifested as shivering, fever, severe hypothermia, respiratory alkalosis, or edema (1). Sepsis can further develop into septic shock, causing multiple organ dysfunction syndrome (MODS) (2). Sepsis in the late stage triggers the immunosuppressive reaction and subsequently leads to persistent or recurrent infection, which has become a primary cause of intensive care unit (ICU) mortality (3). Statistics suggest the incidence rate of sepsis of 0.3% and the mortality rate of 20%-40% (4). A German study of more than 10000 patients from 133 ICUs has shown that the mortality rate of sepsis patients during intensive care is 34.3%. The mortality rate of sepsis patients has a slightly decreasing trend, but the incidence rate of sepsis has increased. It is well-accepted that sepsis exhibits two inflammatory stages: an early dominant hyper-inflammatory phase characterized by the systemic inflammatory response syndrome (SIRS), and the inflammation in this stage can subside; this pro-inflammatory state is followed by an immunosuppressive stage characterized by the occurrence of MODS, also called compensatory anti-inflammatory response syndrome (CARS) (5). Inhibiting the hyper-inflammatory response in the early stage of sepsis can alleviate the damage to immune cells and thus suppress immune cell apoptosis, which is the primary link to preventing and reducing immunosuppression in sepsis (6). Therefore, exploring effective means to alleviate the early inflammatory response is particularly important for the treatment of sepsis.

As the study of epigenetic modifications develops in-depth, the critical role of epigenetic modifications in the early inflammatory response stage of sepsis has been highlighted. Epigenetic modifications refer to the transcription-related modifications on DNA and chromosome while the DNA sequence remains unchanged, which produces heritable phenotypic changes, including RNA methylation, histone modification, and non-coding RNA (7). N^6^-methyladenosine (m^6^A) methylation modification, as the most common and reversible messenger RNA (mRNA) modification, has attracted considerable attention and become a new research hotspot in recent years (8). In addition to mRNA, m^6^A methylation can also modify long non-coding RNA (lncRNA), microRNA (miRNA), ribosomal RNA (rRNA), and transfer RNA (tRNA), etc (9). Existing evidence indicates that m^6^A is involved in the pathogenesis of various diseases by modulating RNA splicing, stability, export, and translation, including tumor, viral infection, pneumonia, and cardiovascular disease (10–12). Importantly, preliminary studies have proven that m^6^A plays an important regulatory role in the immune response of sepsis patients. Herein, this paper summarizes the role of m6A methylation in the inflammatory response of sepsis, with the aim to provide novel insights for further understanding the mechanism of septic inflammatory response and exploring new therapeutic targets.

## 2 Materials and methods

### 2.1 Literature databases

PubMed, China National Knowledge Internet (CNKI), and Wanfang databases were selected for literature retrieval. The literature was retrieved by combining subject words with free words from the database inception to June 30, 2022.

### 2.2 Search terms

#### 2.2.1 English search terms

N^6^-methyladenosine, m^6^A, RNA translation, RNA stability, sepsis, inflammation, and oxidative stress.

#### 2.2.2 Chinese search terms

N^6^-methyladenosine, m^6^A, RNA translation, RNA stability, sepsis, inflammation, and oxidative stress.

## 3 Results

### 3.1 m^6^A methylation

#### 3.1.1 m^6^A methylation modification-related proteins

m^6^A methylation is a ubiquitous mRNA modification in eukaryotes, which can regulate mRNA processing and stability. According to different functions, m^6^A modification proteins can be divided into methyltransferase (writer), demethylase (eraser), and m^6^A-binding protein (reader). For example, methyltransferase-like 3 (METTL3), methyltransferase-like 14 (METTL14), and WT1 proteins can induce RNA methylation, while alkB homolog 5 (ALKBH5) functions as an RNA demethylase that can erase RNA methylation and reverse methylation (13). Moreover, YTH domain family (YTHDF) 1-3 and YTH domain-containing family (YTHDC) 1-2 are m^6^A-binding proteins that recognize m^6^A methylation modification of RNA and thus affect the fate of mRNA (14).

#### 3.1.2 RNA metabolism regulated by m^6^A methylation

m^6^A methylation plays a role in transcription by affecting RNA splicing, export, translation, and stability. Firstly, mRNA is spliced into mature transcripts and exported from the nucleus to the cytoplasm before translation. The process of splicing and export is regulated by m^6^A. Zheng et al. (15) have reported that ALKBH5 can affect mRNA export and metabolism at the same time. After ALKBH5 demethylation, mRNA is mainly concentrated in the nucleus, while in ALKBH5-deleted cells, mRNA is mainly located in the cytoplasm, indicating that ALKBH5 demethylation affects mRNA export. However, knockdown of ALKBH5 can increase the synthesis rate of nascent RNA. Another study has also demonstrated that YTHDC1 is involved in mRNA splicing and export by interacting with exonuclear transporters (16). Secondly, mRNA translation is regulated by m^6^A methylation. YTHDF1 has been reported to promote mRNA translation by binding to m^6^A, while YTHDF1 knockdown leads to a decrease in translation efficiency. YTHDF1 interacts with m^6^A-modified p65 mRNA and facilitates its translation, thereby regulating the inflammatory response (17). Another study by Zhang et al. (18) has shown that YTHDF1 regulates cell stability by promoting the translation of m^6^A-modified Robo3.1 mRNA. In addition, Weng et al. (19) have demonstrated that METTL14 and YTHDF1 participate in neurogenesis by promoting protein translation, implying the pivotal role of m^6^A in mRNA translation. All these findings make m^6^A a new target for inhibiting inflammation. Finally, m^6^A methylation is negatively correlated with RNA stability. Wang et al. (20) have found that the expression of target mRNA is decreased after METTL3 and METTL14 knockdown. YTHDF2 selectively binds m^6^A-methylated mRNA to regulate mRNA degradation (21). In addition, the stability of target mRNA is positively correlated with the number of binding sites after YTHDF2 knockout. Briefly, m^6^A methylation regulates gene expression by affecting RNA metabolism, including splicing and export, degradation, translation, and stability. m^6^A-mediated gene regulation plays a vital role in the pathogenesis of many diseases. Next, the regulatory effects of m^6^A on sepsis progression and septic inflammatory response were discussed.

### 3.2 m^6^A methylation changes in sepsis

#### 3.2.1 Sepsis-induced myocardial injury

In the pathophysiology of sepsis, abnormal inflammation and immune response can aggravate sepsis, leading to shock or myocardial dysfunction (22). Sepsis-induced myocardial injury is a notable factor resulting in poor clinical outcomes in sepsis patients. The heart is one of the most vulnerable target organs in sepsis. Sepsis-induced myocardial injury is mainly manifested by cardiomyocyte degeneration and necrosis, as well as myocardial systolic and diastolic dysfunction (23). The pathophysiological mechanism of sepsis-induced myocardial injury is complex, involving both pathogens and the host immune system. It has also been demonstrated that m^6^A methylation plays a significant regulatory role in sepsis-induced myocardial injury (24). Shen et al. (25) have shown that after endotoxin (0, 0.5, 1, 2, 5, and 10 μg/mL) is applied to the lipopolysaccharide (LPS)-induced cardiomyocyte model for 24 h, the expression of m^6^A methyltransferase METTL3 is elevated with the increase of the dose. Endotoxin can up-regulate the levels of pro-inflammatory cytokines such as interleukin (IL)-6, IL-8, and tumor necrosis factor-α (TNF-α), while silencing METTL3 reduces the levels of pro-inflammatory cytokines. By detecting several m^6^A methylation readers, it is found that the METTL3/insulin-like growth factor 2 mRNA-binding protein 1 (IGF2BP1)/histone deacetylase 4 (HDAC4) axis regulates the inflammatory injury of endotoxin-induced cardiomyocytes. After LPS injection, m^6^A methylation is increased in mouse myocardial tissues, while the expression of fat mass and obesity-associated protein (FTO, an m^6^A demethylase) is decreased correspondingly. These changes are related to the significant increase in the expressions of myocardial inflammatory cytokines, such as IL-6, TNF-α, and IL-1β, as well as the decrease in left ventricular function. In addition, rat myoblasts (H9c2) also present similar changes (enhanced m^6^A RNA methylation, increased expressions of inflammatory cytokines, and decreased FTO gene expression) under LPS treatment (26). In addition, methylated RNA immunoprecipitation assays show that the expressions of IL-6 and TNF-α in endotoxin-treated H9c2 cells are increased compared with those in untreated cells. Rats are allocated to the control group and LPS-induced sepsis group. The m^6^A level of rat left ventricular tissues is determined by liquid chromatography-tandem mass spectrometry (LC-MS), and the transcription level of m^6^A is analyzed by transcriptional exon microarray (mRNAs and lncRNAs). LC-MS-based mRNA modification analysis indicates that after intraperitoneal injection of LPS, the overall m^6^A level in rat aortic tissues is significantly reduced (27). Microarray analysis hints that 40 transcripts (31 mRNAs and 9 lncRNAs) are hypermethylated and 223 transcripts (156 mRNAs and 67 lncRNAs) are hypomethylated in the aortic tissues of LPS-treated rats. The overall m^6^A level in left ventricular myocardial tissues of rats treated with endotoxin is significantly decreased. Similarly, another study unveils the hypermethylation of 27 transcripts (23 mRNAs and 4 lncRNAs) and hypomethylation of 46 transcripts (39 mRNAs and 7 lncRNAs) in the LPS group (28). The mRNA expressions of writers and readers in the LPS group are notably decreased. The m^6^A modification of C-type lectin domain family 1 member B (CLEC1B), serine/threonine kinase 38 like (STK38L), and tumor necrosis factor receptor superfamily member 26 (Tnfrsf26) is related to platelet activation and apoptosis pathways (29). In general, macrophages are activated after the onset of sepsis resulting in the production of large amounts of LPS in the plasma and stimulating the upregulation of m6 A methyltransferase METTL3 expression. Further, METTL3 stimulates enhanced m6A RNA methylation in cardiomyocytes, while increased expression of inflammatory cytokines within the microenvironment leads to cardiomyocyte degeneration and necrosis. The above studies suggest that m^6^A methylation is implicated in the regulation of cardiovascular system damage induced by sepsis.

#### 3.2.2 Sepsis-associated encephalopathy

SAE is a serious brain dysfunction mainly caused by sepsis in the non-central nervous system, with clinical manifestations of consciousness disorder, mild cognitive impairment, insanity, and coma. The mortality rate of SAE is as high as 30%-70%, which seriously compromises the health of patients (30). Currently, electroencephalogram, transcranial Doppler, and a series of serum markers including intercellular adhesion molecule-5 (ICAM-5) and S-100β have a certain value in the early diagnosis, evaluation, and prognosis of SAE (31). The specific pathogenesis of SAE has not been fully elucidated. The occurrence of SAE is thought to be related to nonspecific inflammation and non-inflammatory responses of brain cells. Changes in the metabolic function of brain cells after brain injury underlie the pathogenesis of SAE. Moreover, neuroinflammation is the main mechanism of m^6^A methylation recently reported to be associated with the occurrence of SAE (32). Previous studies have shown that the levels of white blood cell (WBC), neutrophil (Neut), platelet (PLT), and IL-6 in the SAE group are higher than those in the non-SAE group. Spearman correlation coefficient analysis has indicated a significant correlation between intestinal flora/serum metabolites and SAE. The number of Acinetobacter is positively correlated with the expression of METTL3. Thus, these findings further reveal the regulatory axial relationship of intestinal tract-methylation-inflammation-SAE (33).

Due to the increasing number of studies based on the gut-brain axis, existing mechanistic studies have introduced metabolic changes induced by the gut microbiome into the genesis of SAE. The gut-brain axis includes the central nervous system, the central endocrine system and the central immune system, which includes the hypothalamic-pituitary-adrenal axis (HPA axis), the sympathetic, parasympathetic (vagal) and enteric nervous systems of the autonomic nervous system, and the microbiota in the gut. The gastrointestinal surface is mainly governed by the autonomic nervous system, but the stimulation of the gastrointestinal tract’s bacteriophage also induces the secretion of physiological regulatory messages by the gut epidermal cells, which not only induce local immune responses, but also transmit physiological messages to the brain center *via* the autonomic nervous system, which in turn affects the central endocrine system and the central immune system. The pathogenic route based on the intestinal flora has been first investigated, namely the expression of METTL3 in immune cells stimulated by abundant immobile metabolites. However, studies on SAE-induced methylation are still in their infancy and further in-depth studies and explorations are still needed to determine the specific mechanisms.

#### 3.2.3 Sepsis-associated acute kidney injury

The lung is particularly susceptible to sepsis injury (34). About 50% of patients with severe sepsis may develop acute respiratory distress syndrome (ARDS), with a mortality rate of 30%-40%. Unfortunately, no specific treatment has been proven to be effective against sepsis-caused ARDS so far (35). Further investigation on the pathogenesis of SA-AKI can provide novel treatment strategies for this urgent clinical issue, thus reducing ICU mortality. Many genetic and epigenetic alternations are related to the maintenance of endothelial function (36). There is increasing evidence that sepsis-related pulmonary microvascular leakage and organ damage/dysfunction are associated with changes in epigenetics and gene expression (37). Chemical modification of RNA molecules is crucial for the post-transcriptional regulation of gene expression. m^6^A is the most abundant form of mRNA and lncRNA methylation in eukaryotic cells, affecting a variety of physiological and pathological processes. YTH family proteins, IGF2BP, and heterogeneous nuclear ribonucleoprotein A2/B1 (hnRNPA2/B1) have been confirmed to affect mRNA stability and translation (38). Abnormal m^6^A methylation is responsible for the occurrence and development of human diseases, suggesting the vital role of m^6^A modification in different genomic backgrounds. Chen et al. (39) have found that in sepsis, the reduction of m^6^A modification is associated with the down-regulation of METTL3. METTL3 knockout can accelerate barrier dysfunction and inflammatory response. In addition, tripartite motif containing 59 (TRIM59) is considered to be a key m^6^A effector, and its deletion exacerbates lung injury. Mechanistically, m^6^A reader YTHDF1 recognizes and stabilizes m^6^A-modified TRIM59 mRNA to protect vascular endothelial cells from barrier dysfunction and inflammatory response (40).

Wang JN et al. reported that METTL3 expression was elevated in renal tubules from different AKI models as well as in human biopsied and cultured tubular epithelial cells (TECs) (41). The researchers evaluated the specific function of METTL3 in HK2 cells in response to inflammatory stimuli. The results showed that silencing METTL3 attenuated renal inflammation and programmed cell death in TECs in response to TNF-α (tumor necrosis factor-α), cisplatin and LPS (lipopolysaccharide) stimulation, whereas METTL3 overexpression had the opposite effect. Conditional knockout of METTL3 in mouse kidneys attenuated cisplatin and ischemia/reperfusion (I/R)-induced renal dysfunction, injury and inflammation. This experiment reveals the mechanism of action by which genetic and pharmacological inhibition of METTL3 attenuates TAB3 m6A modification through an IGF2BP2-dependent mechanism, thereby reducing renal injury and inflammation, suggesting the METTL3/TAB3 axis as a potential target for the treatment of AKI.

### 3.3 m^6^A methylation and immune cells in sepsis

#### 3.3.1 Neutrophils

**Figure 1:**
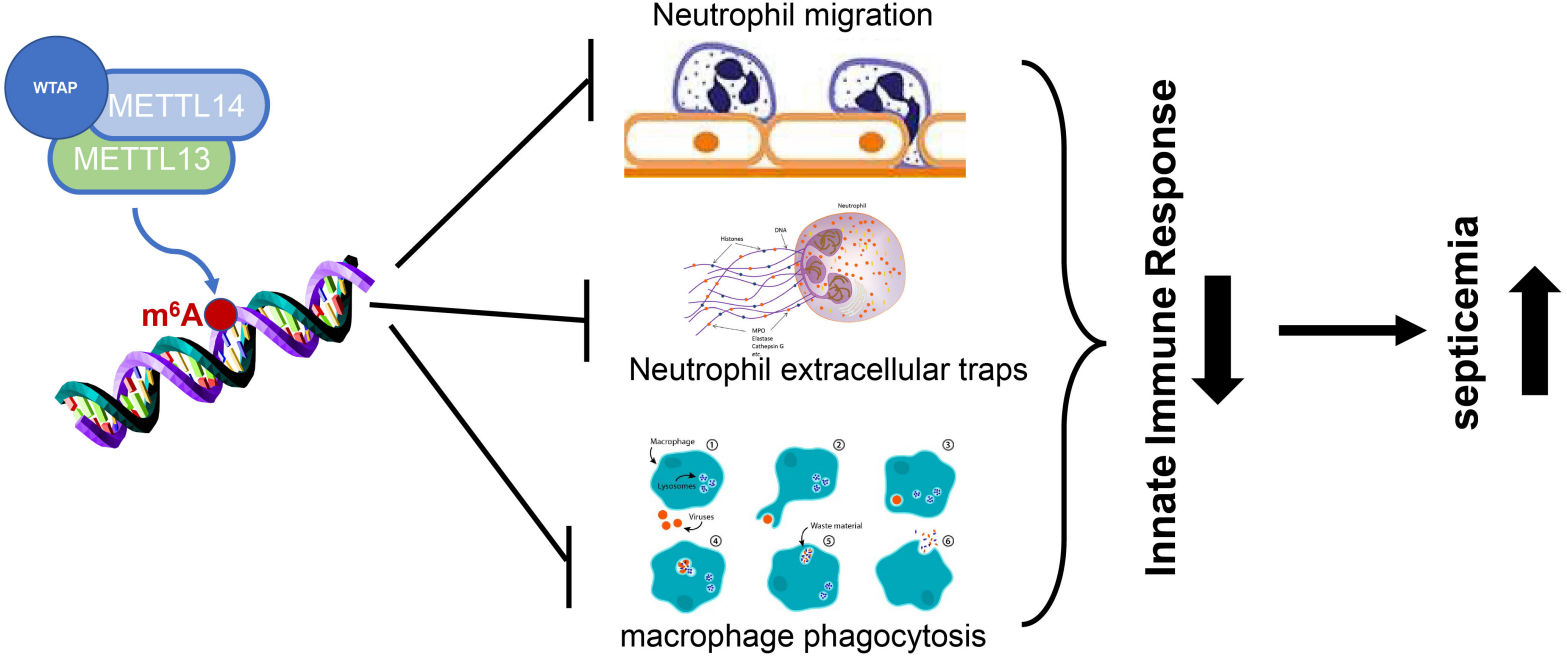
Schematic representation of the possible mechanisms of m6A methylation leading to sepsis.

Neutrophils are the primary components of innate immune response and also the main population of leukocytes in peripheral blood, functioning as important innate defense cells of the host against invading pathogens. During the bacterial attack, a large number of neutrophils are mobilized and released from the bone marrow into the peripheral circulation and migrate to the infection site at a very early stage (42). The rapid and effective migration and aggregation of neutrophils at the site of infection are essential for the innate defense against bacteria and the subsequent regression of inflammation (**Figure 1**). The migration and function of neutrophils can be realized by extracellular signals such as chemokines and cytokines, and also by the intrinsic elements of neutrophils including chemokine receptors, cytoskeleton proteins, intracellular signal transduction modes, and cell metabolism (43). As the first line of the innate immune response, once the migration of neutrophils is impaired due to the imbalance of the above processes, the host cannot initiate effective innate defense against bacterial infection in a timely manner, resulting in failure of bacterial clearance, systemic inflammation, and even sepsis. On the other hand, bacterial infection may hijack the migration of neutrophils to escape the innate defense of the host, leading to persistent infection and unresolved inflammation (44). Therefore, the identification of new intracellular molecules that essentially determine the migration ability of neutrophils can not only enhance the innate defense against bacteria in the early stage of infection but also appropriately induce inflammatory resolution to avoid tissue damage after the removal of invading bacteria. ALKBH5 deficiency increases the mortality of mice with polybacterial sepsis induced by cecal ligation and perforation (CLP), and ALKBH5-deficient CLP mice exhibit higher bacterial load and more pro-inflammatory cytokines in the peritoneal and blood due to reduced neutrophil migration (45). ALKBH5-deficient neutrophils have lower C-X-C motif chemokine receptor 2 (CXCR2) expression and thus exhibit impaired migration to C-X-C motif chemokine ligand 2 (CXCL2). ALKBH5-mediated m^6^A demethylation targets neutrophil migration-related molecules by altering RNA decay to enhance the migration ability of neutrophils, such as increasing the expressions of neutrophil migration-promoting CXCR2 and NLR family pyrin domain containing 12 (NLRP12), but decreasing the expressions of neutrophil migration-suppressive prostaglandin E receptor 4 (PTGER4), tenascin-C (TNC), and with-no-lysine kinase 1 (WNK1) (46).

Xuetao Cao’s group at Peking Union Medical College reported that ALKBH5-mediated m6A demethylation enabled neutrophils to have high migratory capacity by altering RNA decay, thereby regulating the protein expression of their targets, neutrophil migration-related molecules, including increased neutrophil migration promoting CXCR2 and NLRP12 expression, but decreased neutrophil expression migration inhibiting PTGER4, TNC and WNK1 (47). In conclusion, the results of this study reveal an unknown role for ALKBH5 in imprinting neutrophil migration-promoting transcriptome features and intrinsically promoting neutrophil migration for antimicrobial defense, highlighting the potential application of targeting neutrophil m6A modifications in the control of bacterial infections.

The generation of neutrophil extracellular traps (NETs) is the main strategy of polymorphonuclear neutrophils (PMN) against microorganisms. NETs are closely related to the pathogenesis of various lung injuries, but relatively little is known about the role of NETs in SA-AKI (37). Elevated levels of NETs and ferroptosis can be observed in infected ARDS patients and SA-AKI mice. Blocking the formation of NETs by peptidyl arginine deiminase 4 (PAD4) inhibitor and PAD4 knockdown in mice, or inducing the depletion or degradation of NETs by anti-Ly6G and DNase, can significantly reduce sepsis-induced ferroptosis and protect mice from SA-AKI. Moreover, the cellular damage caused by NETs to human epithelial cells is weakened when ferroptosis is blocked. RNA-seq and qPCR analysis have shown that the expression of METTL3 is significantly increased during the ferroptosis of alveolar epithelial cells induced by NETs (47). *In vitro* models of METTL3 knockout and overexpression elucidate that METTL3-mediated m^6^A modification of glutathione peroxidase 4 (GPX4) is involved in the ferroptosis of human alveolar epithelial cells induced by NETs. The *in vivo* model of CLP-induced sepsis and the murine model of SA-AKI using METTL3 KO mice further demonstrate the critical role of METTL3-mediated m^6^A modification in NET-induced ferroptosis and protection of mice from SA-AKI (48).

#### 3.3.2 Macrophages

Recent studies have established the critical role of macrophages in the pathogenesis of sepsis-caused ARDS (49) (**Figure 1**). Macrophages can change from the initial pro-inflammatory M1 phenotype at the onset of lung injury to the anti-inflammatory M2 phenotype at the stage of initiating lung repair (50). Direct evidence has been provided to demonstrate the ability of polarized M2 phenotype *in vivo* and *in vitro* to prevent lung injury, inflammation, and subsequent fibrosis in ARDS (51). Zhao et al. have explored the differential expression pattern of circular RNAs (circRNAs) in pulmonary macrophages of sepsis-induced ARDS mice using microarray and found that circN4bp1 affects macrophage differentiation by binding to miR-138-5p, thereby regulating enhancer of zeste homolog 2 (EZH2) expression *in vivo* and *in vitro*. Subsequently, elevated m^6^A levels of circN4bp1 are found in ARDS mice, and inhibition of m^6^A methyltransferase METTL3 reverses this trend *in vitro* (52). Therefore, circN4bp1 can act as a sponge of miR-138-5p and modulate the polarization of macrophages by regulating the expression of EZH2. YTHDF1 knockdown improves the immune paralysis of macrophages, Th1/Th17 cells, and cytotoxic T lymphocytes (CTLs) and also alleviates macrophage-caused endothelial damage in severe sepsis rats with extracorporeal membrane oxygenation (ECMO) by suppressing the high mobility group box-1 (HMGB1)/receptor for advanced glycation end products (RAGE) axis and YTHDF1 and decreasing the m^6^A RNA methylation of Janus kinase 2 (JAK2)/signal transducer and activator of transcription 3 (STAT3) and pJAK2/pSTAT3 proteins in macrophages (53).

There is also compelling evidence that epigenetic regulation coordinates dynamic macrophage polarization. m6A methylation is the most abundant epigenetic modification in species mammalian mRNAs, but its role in macrophage polarization remains completely unknown. m6A catalase METTL3 was shown to be specifically upregulated by Vu LP et al. following M1 polarization in mouse macrophages (54). Furthermore, knockdown of METTL3 by siRNA transfection significantly inhibited M1 but enhanced M2 macrophage polarization. Conversely, its overexpression by plasmid transfection greatly promoted M1 but attenuated M2 macrophage polarization. Further immunoprecipitation of methylated RNA and *in vitro* m6A methylation analysis showed that METTL3 directly methylates mRNA encoding STA T1, the master transcription factor controlling M1 macrophage polarization, located in its CDS and 3’UTR regions. Furthermore, METTL3-mediated methylation of STAT1 mRNA significantly increased mRNA stability and subsequently upregulated STAT1 expression. In conclusion, METTL3 drives M1 macrophage polarization through direct methylation of STAT1 mRNA and may serve as an anti-inflammatory target.

#### 3.3.3 NK cells

In recent years, more and more scientists have focused on the role of m6A methylation modifications in the immune system. However, the effect and regulation of this methylation on NK cells is not yet clear. Recently, a researcher has studied this. The results showed that the expression of the m6A “writer” METTL3 was decreased in tumor-infiltrated NK cells, and the protein expression level of METTL3 was positively correlated with the effector molecules in NK cells.

METTL3 deficiency in NK cells alters NK cell homeostasis and inhibits NK cell infiltration and function in the microenvironment. The gene encoding SHP-2 is modified by m6A and its protein expression is reduced in METTL3-deficient NK cells, which is associated with the suppression of AKT and MAPK signaling pathway activation in mettl3-deficient NK cells (55).

Overall, macrophages are activated after the onset of sepsis resulting in the production of large amounts of LPS in the plasma and stimulating the upregulation of m6 A methyltransferase METTL3 expression. Further, METTL3 stimulates enhanced m6A RNA methylation in cardiomyocytes, while increased expression of inflammatory cytokines within the microenvironment leads to cardiomyocyte degeneration and necrosis.

## 4 Summary and prospect

m^6^A methylation modification is not only necessary for RNA metabolism but also a pivotal factor for maintaining normal cell function. Aberrant alternations in m^6^A methylation can further exacerbate inflammatory responses in sepsis patients and ultimately accelerate disease progression and organ function damage. Therefore, the role of m^6^A methylation in sepsis deserves the attention of researchers. However, the existing research still has some limitations. It remains elusive whether m^6^A modification-related proteins play a synergistic or antagonistic role in the same process. There are few studies on m^6^A methylation at present, and its exact regulatory mechanism is still unclear to a large extent. Hence, further basic and clinical studies are warranted.

## Data availability statement

The original contributions presented in the study are included in the article/supplementary material. Further inquiries can be directed to the corresponding author.

## Author contributions

WQ and YC designed the study. WQ and YC provided methodology. WQ and YC performed the formal analysis. WQ and YC wrote and revised the manuscript. WQ and YC supervised the entire study. All authors contributed to the article and approved the submitted version.

## Acknowledgments

We would like to acknowledge the reviewers for their helpful comments on this paper.

## Conflict of interest

The authors declare that the research was conducted in the absence of any commercial or financial relationships that could be construed as a potential conflict of interest.

## Publisher’s note

All claims expressed in this article are solely those of the authors and do not necessarily represent those of their affiliated organizations, or those of the publisher, the editors and the reviewers. Any product that may be evaluated in this article, or claim that may be made by its manufacturer, is not guaranteed or endorsed by the publisher.
